# Longitudinal Typhoid Fever Trends in India from 2000 to 2015

**DOI:** 10.4269/ajtmh.18-0139

**Published:** 2018-07-25

**Authors:** Veeraraghavan Balaji, Arti Kapil, Jayanthi Shastri, Agila Kumari Pragasam, Geeta Gole, Sirshendu Choudhari, Gagandeep Kang, Jacob John

**Affiliations:** 1 Christian Medical College, Vellore, Tamil Nadu, India;; 2 All India Institute of Medical Sciences, New Delhi, India;; 3 TN Medical College & B Y L Nair Hospital, Mumbai, Maharashtra, India;; 4 Translational Health Sciences Technology Institute, Faridabad, Haryana, India

## Abstract

A very high incidence of typhoid was described in studies conducted in urban locations on the Indian subcontinent at the end of the twentieth century. Despite their availability, licensed immunogenic conjugate typhoid vaccines have not been introduced in the national immunization program, in part, because of a lack of understanding of where and for whom prevention is most necessary. Uncertainty regarding the burden of disease is based on the lack of reliable, recent estimates of culture-confirmed typhoid and an observed trend of low isolations of *Salmonella* Typhi and fewer complications at large referral hospitals in India. In this article, we examine the trends of *S.* Typhi isolation at three large tertiary care centers across India over 15 years and describe trends of recognized risk factors for typhoid from published literature. There appears to be a decline in the isolation of *S.* Typhi in blood cultures, which is more apparent in the past 5 years. These trends are temporally related to economic improvement, female literacy, and the use of antibiotics such as cephalosporins and azithromycin. The analysis of trends of culture-confirmed typhoid may not accurately capture the typhoid incidence trends if antibiotic use confounds the burden of disease presenting to larger facilities. Emerging antimicrobial resistance may result in a resurgence of disease if the underlying incidence and transmission of typhoid are not adequately addressed through public health approaches.

## INTRODUCTION

The global incidence of typhoid fever has been estimated to be between 11.9 and 26.9 million cases.^1,2^ Typhoid fever is believed to be responsible for an estimated 129,000–161,000 deaths each year, with a large proportion of these in South Asia.^1,3^ Although typhoid is believed to be endemic in Asia, an estimation of the magnitude of burden has been confounded by the lack of access to laboratories in settings that are most likely to have a high disease burden and the poor diagnostic accuracy of available tests.

The Diseases of the Most Impoverished projects between 2000 and 2006 helped quantify the disease burden of typhoid and paratyphoid across different settings in Asia. These population-based studies in the urban slums of India, Pakistan, and Indonesia found the incidence of blood culture–confirmed typhoid to be between 180 and 494 cases per 100,000 children aged 5–15 years.^4^ Younger children between 2 and 4 years were noted to be at higher risk with incidence rates between 149 and 573 cases per 100,000 children.^4,5^ A prospective cohort study in the early 1990s in the slums of Delhi revealed an incidence of culture-confirmed typhoid of 980 cases per 100,000 population per year with a much higher incidence of 2,730 cases per 100,000 child years in children younger than 5 years.^6,7^ Similar findings of high disease burden in younger children was noted in a study from an urban slum in Bangladesh in 2001 where the incidence was 1,870 cases per 100,000 pre-school children per year.^8^ These studies indicated that despite heterogeneity in estimates from different settings, India had a significant burden of typhoid especially among its young children.

A recent systematic review on the typhoid burden in India in 2016 estimated the prevalence of laboratory-confirmed typhoid and paratyphoid among individuals with fever across all hospital studies at 9.7% (95% confidence interval: 5.7–16.0%) and 0.9% (0.5–1.7%), respectively. This systematic review noted a significant decline in the prevalence of typhoid fever in recent years in the multivariate meta-regression model.^9^ The apparent decline in culture-confirmed typhoid and the lack of complications such as intestinal perforation at tertiary care centers over the past decade has resulted in a prevalent view among tertiary care clinicians that typhoid incidence is declining.

Multidrug-resistant (MDR) *Salmonella* Typhi, defined as strains resistant to ampicillin, chloramphenicol and cotrimoxazole developed and increased in frequency from the 1990s. Following the increase in MDR *S.* Typhi, treatment practices shifted to the use of fluoroquinolones.^10–13^ However, over the past 30 years, there have been reports of continuous transmission of H:58 clade of *S.* Typhi in Asian and African countries.^14^ This clade is usually associated with fluoroquinolone resistance and is a global threat to the effective treatment of typhoid. Our current data from India on this strain are limited but resistance to ciprofloxacin is almost universal in recent Indian isolates. Presently, azithromycin and ceftriaxone remain good choices for treatment, with *S.* Typhi isolates showing limited evidence of clinical or in vitro resistance. It is also important to note that the antibiotics to which *S.* Typhi isolates were resistant during the 1990s and early 2000s viz. ampicillin, chloramphenicol, and co-trimoxazole; are now likely to be effective as recent isolates have become increasingly susceptible.^15^

## METHODS

We reviewed laboratory data from three large medical centers located in geographically distinct settings across India to examine trends in *S.* Typhi isolation, antimicrobial susceptibility and to examine factors that potentially contributed to these trends. We reviewed blood culture records at the Christian Medical College (CMC), Vellore; All India Institute of Medical Sciences (AIIMS), New Delhi; and B Y L Nair (BYLN) Hospital, Mumbai, between 2000 and 2014. There were no significant changes in the culture methods or diagnostic algorithms at these sites during the study period although antimicrobial susceptibility testing followed the current Clinical and Laboratory Standards Institute guidelines. Where available, the data were categorized by age and outpatient or inpatient status. The only difference in the methodology is the revision of ciprofloxacin interpretation criteria in the year 2012 by the CLSI guidelines. The total number of blood cultures performed annually, the number of cultures that grew any clinically significant organism (non-contaminants), and the number of *S.* Typhi– and *S.* Paratyphi–positive cultures was recorded. For trend analysis, the frequency of *S.* Typhi isolates and the proportion of blood cultures with *S.* Typhi in each calendar year were obtained.

To examine contextual factors that could influence secular trends, we reviewed data on access to safe water, hygiene and sanitation, population density, and economic growth from nationally representative demographic health surveys, and monitored reports by United Nations Children’s Fund (UNICEF), the World Bank, and the Joint Monitoring Program for Water Supply and Sanitation. We also reviewed data on antimicrobial use and antimicrobial resistance in isolates using published data from the region and provided descriptive analyses of trends of typhoid fever and its known determinants.

## RESULTS

**Figure 1.**
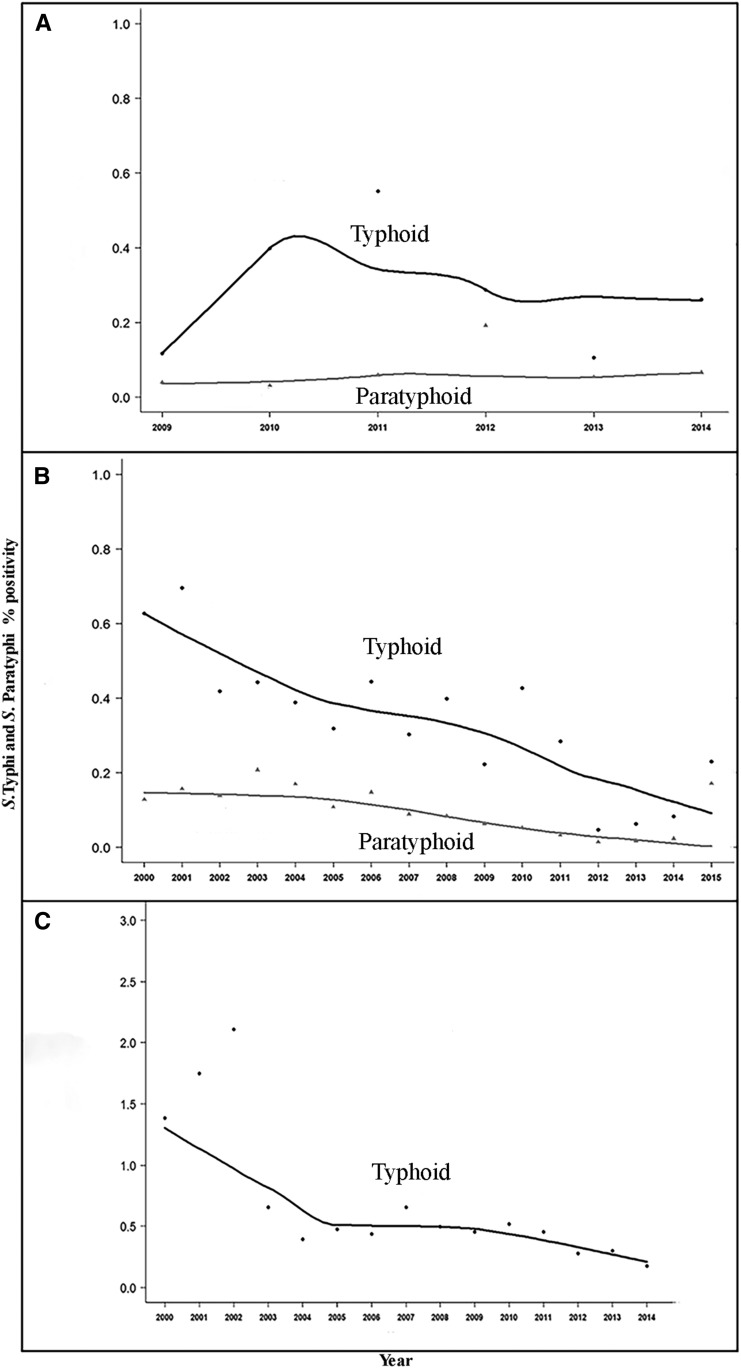
(**A**) Trends in *Salmonella* Typhi and *Salmonella* Paratyphi isolation at B Y L Nair Hospital, Mumbai. (**B**) Trends of *S.* Typhi and S. Paratyphi at All India Institute of Medical Sciences, New Delhi. (**C**) Trends of *S.* Typhi isolation at Christian Medical College, Vellore.

All three hospitals had broadly similar patterns of isolation of *S.* Typhi in blood culture (Figure 1). The number of annual isolates declined from 220 in CMC Vellore in 2000 to 98 in 2015. The trends in CMC, Vellore, were characterized by a steep decline between 2001 and 2004 with a gradual increase thereafter until 2010 followed by a remarkable decline in *Salmonella* Typhi isolation. In AIIMS, New Delhi, the trend showed a decline from 103 isolates in 2000 to 31 isolates in 2015, with a more accelerated decline after 2010 that mirrors the decline in Vellore. The data from BYLN, Mumbai, reveals a similar decline between 2010 and 2015. The proportion of blood cultures that resulted in positive *S.* Typhi isolates declined from 0.62% in 2000 to 0.18% in 2015 in AIIMS, whereas in CMC, it declined from 1.38% in 2000 to 0.17% in 2015. In BYLN, 0.4% of blood cultures were positive for *S.* Typhi in 2010 and this declined to 0.2% in 2015. These trends suggest that the isolation of *S.* Typhi declined at all three sites and the decline was most pronounced in the last 5 years (Figure 1).

### Patterns of antimicrobial resistance.

Antimicrobial susceptibility profiles published from India with *S.* Typhi and *S.* Paratyphi collected between 2001 and 2013 were analyzed. In *S.* Typhi, ampicillin resistance ranged from 3.7% to 72%, with significant regional variations. Notably, the resistance rates decreased to 6–18% in 2014. For co-trimoxazole, resistance rates ranged from 2.5% to 35% between 2001 and 2013. Recently, resistance to co-trimoxazole was reported to be 3.7% and 23.4% with significant regional variation. For chloramphenicol, resistance ranged from 2.5% to 34% during 2001–2008, with a decline to < 5% since 2010. Overall, the resistance rates for ampicillin, chloramphenicol, and co-trimoxazole have declined recently, as resistance to more widely used antibiotics has risen. Among the fluoroquinolones, nalidixic acid resistance was widely reported from the 1990s, ranging during the period of this report from 76% to 100%, in different regions of India. In 2015, the CLSI guidelines were revised to include pefloxacin disc diffusion testing for quinolone susceptibility. Initially, ciprofloxacin resistance ranged from 1% to 26%, and in more recent testing, resistance rose to 98%, using the revised CLSI 2012 guidelines interpretative criteria. Of all the antimicrobials tested, ceftriaxone was the most active agent, with resistance rates of about 1.5–4%. As presented in Table 1, very few studies have reported ceftriaxone resistance in India, leaving this drug a promising choice for empirical therapy for severe typhoid cases. Cefixime resistance is also very low at 0.2–2%, reported in only one study from India (Table 1).

**Table 1:** Antimicrobial resistance in *Salmonella* Typhi and *Salmonella* Paratyphi from Indian studies^11,16–24^

Reference		Year of isolates	Ampicillin	Chloramphenicol	Co-trimoxazole	Nalidixic acid	Ciprofloxacin	Cefixime	Ceftriaxone
Singhal et al. (2014)^20^	*S.* Typhi	2001–2012	< 5%	< 5%	< 5%	100%	5.8–18.2% 97.7% as per new criteria (2012)	0%	0%
Dutta et al. (2014)^19^	*S.* Typhi	2009–2013	18.2%	22.1%	23.4%	98.7%	19.5%	–	–
Jain and Chugh (2013)^27^	*S.* Typhi	2010–2012	3.7–6.5%	2.7–4.6%	0–3.7%	93.5–100%	–	0.9–2%	0–2%
Raza et al. (2012)^18^	*S.* Typhi	2010–2011	–	6.4%	–	–	0%	–	4.3%
Shetty et al. (2012)^17^	*S.* Typhi	2009–2011	5.89%	3.53%	2.35%	81.18%	3.53%	–	0%
Menezes et al. (2011)^16^	*S.* Typhi	2005–2009	34.1%	34.1%	34.1%	78%	8%	–	0%
Muthu et al. (2011)^28^	*S.* Typhi	2007–2009	32.5%	2.5%	2.5%	96.25%	6.5%	–	1.5%
Bhattacharya et al. (2011)^11^	*S.* Typhi	2005–2008	21.36%	8.97%	27.35%	–	1.71%	–	3%
Dutta et al. (2014)^19^	*S.* Paratyphi A	2009–2013	0%	0%	0%	96%	20%	–	–
Jain and Chugh (2013)	*S.* Paratyphi A	2010–2012	3–6.2%,	0%	0%	100%	–	0–6.2%	0–6.2%
Raza et al. (2012)^18^	*S.* Paratyphi A	2010–2011	74.2%	0%	0%	–	0%	–	0%
Shetty et al. (2012)^17^	*S.* Paratyphi A	2009–2011	18.75%	0%	0%	62.5%	18.75%	–	6.25%
Muthu et al. (2011)^28^	*S.* Paratyphi A	2007–2009	–	5%	5%	–	12.5%	–	4.5%
Bhattacharya et al. (2011)^11^	*S.* Paratyphi A	2005–2008	28.12%	23.44%	35.94%	–	1.56%	–	4.69%

For *S.* Paratyphi, the antimicrobial susceptibility profile reported across India during 2005 and 2013 were analyzed. For ampicillin, co-trimoxazole, and chloramphenicol, the resistance rates ranged from 3–28%, 5–36%, and 5–23%, respectively. Among the fluoroquinolones, 1.5–20% resistance was reported for ciprofloxacin. Similar to *S.* Typhi, high nalidixic acid resistance was noted, ranging from 63% to 100%. By contrast to *S.* Typhi, slightly higher resistance to ceftriaxone was reported in *S.* Paratyphi ranging from 4.5% to 6.2% across various regions in India. One report showed cefixime resistance to be 6.2% in *S.* Paratyphi. Overall, from the susceptibility profiles, over time, declining MDR rates were seen.

Trend analysis between the year 2000 and 2015 at Vellore showed a decline in the resistance rates from about 50% to < 5% for the first line agents such as ampicillin, chloramphenicol, and co-trimoxazole for *S.* Typhi. By contrast, resistance was < 10% in *S.* Paratyphi over the 15 years for ampicillin, chloramphenicol, and co-trimoxazole. Notably, ciprofloxacin resistance in both *S.* Typhi and *S.* Paratyphi was < 10% until 2011. However, the revised ciprofloxacin breakpoints by CLSI guidelines resulted in much higher resistance rates of about 88% to 97% in 2012 and 2015, respectively. This observation was similar for both *S.* Typhi and *S.* Paratyphi. Nalidixic acid resistance was > 80% throughout the study period.

Although ceftriaxone remains the most active agent for typhoidal *Salmonella* isolates, with susceptibility of almost 100% over the past 15 years, ceftriaxone resistance was observed for the first time in 2011 in about 2.4% and 3.4% isolates of *S.* Typhi and *S.* Paratyphi, respectively at CMC. Usage of antibiotics and resistance patterns matched with each of the hospital data analyzed.

### Trends in contextual factors.

According to the UNICEF and World Health Organization (WHO) Joint Monitoring Program, significant strides have been made in improving drinking water and sanitation in India with access to improved sanitation in urban areas gradually increasing from 49.3% in 1990 to 62.6% by 2015. In rural areas, the change was from 5.6% to 28.5% over the same time-period. Access to piped water increased from 47% in urban settings in 1990 to 54% in 2015 and in rural areas from 6% in 1990 to 16% by 2015.^25^ The estimated number of individuals practicing open defecation has decreased marginally from 653 million in 1990 to an estimated 569 million in 2015.^26^ The improvements in access to safe water and improved sanitation facilities have not been uniform across the country, with large differences at state and district levels.

An expanding population has resulted in the population density increasing from 298.8 people per square kilometer (KM) in 1991 to 440.9 per square KM in 2015, but with improvements in urban planning, the proportion of the urban population living in the slums has decreased from 54.9% in 1990 to 24% in 2015. Concerted efforts in the past decade have led to improved female literacy with the World Bank estimating that the adult female literacy rate increased from 33.7% in 1991 to 63.0% in 2015. The gross national income (GNI) in 1991 was United States Dollars (USD) 314.1 billion and rose marginally to USD 450 billion by 2000. Subsequent economic growth resulted in a dramatic increase in GNI to USD 2.1 trillion in 2015. Similarly, the per capita gross domestic product (GDP) rose substantially from USD 309 in 1990 to USD 1,598 in 2015. The public spending on health remained relatively unchanged between 1.05% and 1.16% of GDP between 1995 and 2010 but increased thereafter to 1.41% in 2014 (Table 2).^26^

**Table 2:** Trends in contextual factors in India between 1991 and 2015

		1991	1995	2000	2005	2011	2014	2015
Improved sanitation (%)	Urban	49.3	51.6	54.5	57.4	60.3	62.6	62.6
	Rural	5.6	9.6	14.5	19.5	24.5	28.5	28.5
Open defecation (million)*		653	667	660	638	602	576	569*
Adult female literacy rate (%)		33.7	–	47.8	50.8	59.3	–	63.0
Population density (per square km)		298.8	323.1	360.5	384.8	419.5	435.7	440.9
% Urban population living in slums		54.9	48.2	41.5	34.8	29	24	–
Gross national income (in billion USD)		314.01	360.6	475.4	830.1	1,715	2,012	2,092
GDP per capita in current USD		309	381	460	730	1,455	1,577	1,598
Government spending on health (% of GDP)		–	1.05	1.07	1.13	1.16	1.41	–
Cephalosporin use (standard units per 1,000 population)		–	–	1,939	3,277	6,751	7,269	–

GDP = gross domestic product.

* Projected.

**Figure 2.**
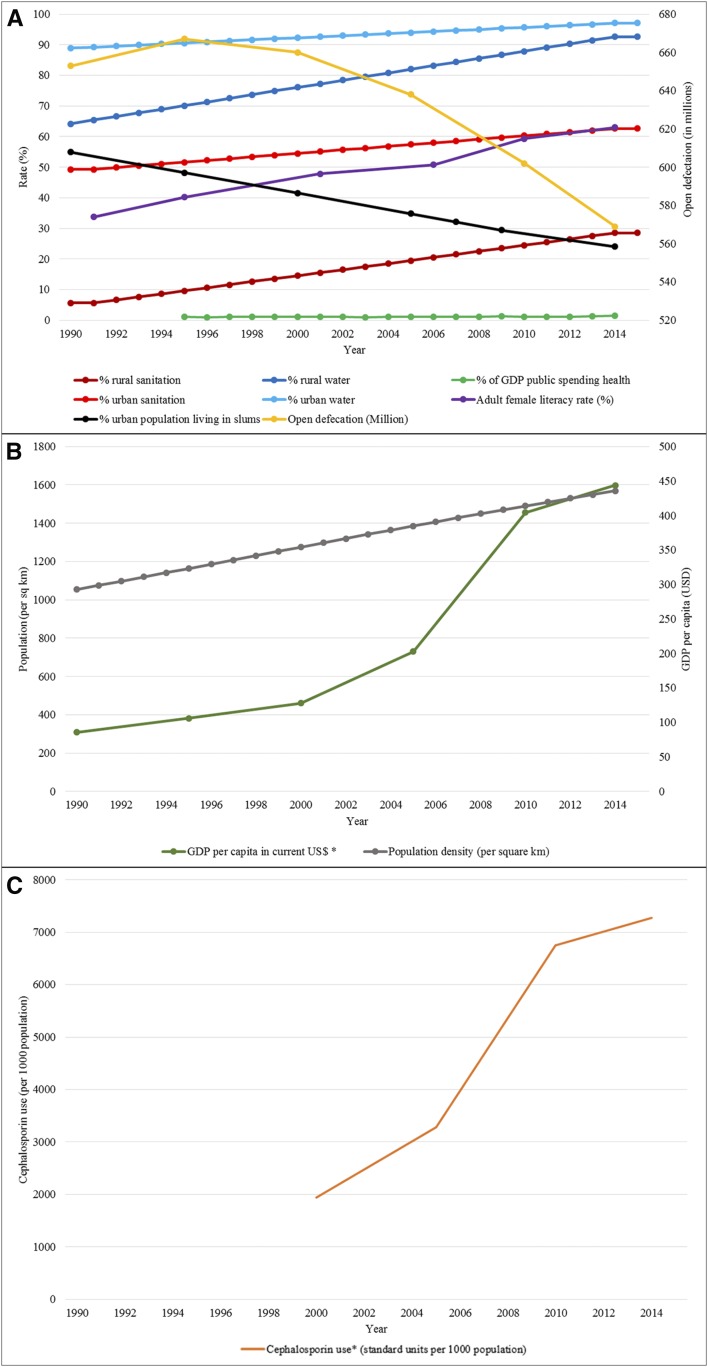
Trends in contextual factors. Panel **A** shows trends in access to improved sanitation, improved water supply, %GDP spending, adult female literacy rate, % of population living in urban slums, and open defecation. Panel **B** shows the trend in population density and GDP per capita. Panel **C** shows the use of cephalosporin in standard units per 1,000 population. GDP = gross domestic product.

Details of the immunization coverage with typhoid vaccines is unavailable but the uptake of typhoid vaccines is limited to the private market in most of the country with only Delhi state having a school-based vaccination program from 2000. Per IMS Health data on the Center for Disease Dynamics, Economics & Policy website, antibiotic use, especially cephalosporins and macrolides, have increased significantly between 2001 and 2014. Cephalosporin use has increased from 1939 standard units per 1,000 population to 7,269 in 2014 (Table 2).^29^ The use of antibiotic combinations has increased in the past decade, as has the availability of azithromycin which is off-patent and available at low prices.^29–31^ Contextual factor trends are shown in Figure 2.

## DISCUSSION

Typhoid, which was widely prevalent across the world has been effectively controlled in many developed countries, likely through large-scale water treatment projects and establishing sewerage systems. Our review of the trends suggests that although commendable, the progress in access to safe water and sanitation is neither qualitatively nor quantitatively sufficient to explain the decline in typhoid across different settings in India. Large-scale water and sanitation community trials in India have had only moderate impact in decreasing fecal contamination of water and prevention of enteric diseases.^32^ In large parts of India, improved water measured by global indicators does not translate to access to safe water. Access to safe water and sanitation remains inadequate because of the considerable resources required to establish and maintain these systems. Although these systems will eventually be established, it is likely to take several years, if not decades, to create an environment that provides an effective barrier to *S.* Typhi. The costs of universal access were approximately USD 35 billion per year for sanitation and USD17.5 billion for drinking water per year from 2010 to 2015.^33^ Densely populated, poorly planned and unregulated urban settlements have very little space for public utilities and pose logistic challenges to creating de-novo water and sewerage systems, resulting in inequitable distribution of these systems with people at highest risk of disease having the least access. Other interventions such as hygiene education, use of soap, safe excreta disposal, and regulating commercial food handling incur lower costs and are, therefore, easier to implement.^34^

There has been an overall improvement in economic conditions and a decline in the proportion of people in urban slums in India. Although part of the decline in the proportion of people living in the slums could be explained by variations over time of how slums have been defined, these factors have the potential to impact both access to health care and the probability of developing enteric fever. Economic improvements in the recent past have mirrored the period of greatest decline in typhoid bacilli isolation. Improvements in economic conditions have resulted in more disposable income and early access to health care. In addition, the industrial growth and expiry of critical drug patents have made access to life-saving antibiotics cheaper and more accessible to large parts of the population. The use of cephalosporins and macrolides has increased significantly and along with improvements in GNI represent the most dramatic change in contextual factors. The rampant, early and indiscriminate use of antibiotics provides a possible explanation for the observed typhoid isolation trends. Antibiotics can lead to a decline in culture-confirmed typhoid in hospitals by decreasing the probability of a typhoid episode being identified by culture. Early antibiotic therapy can reduce the quantum and duration of convalescent carriage and the incidence of complications. Antibiotics are more likely to explain the remarkably similar trends in typhoid isolation at geographically distinct health facilities than improvement in sanitation and hygiene that are more likely to be focal. However, the emergence of drug-resistant strains in the future may unmask clinical infection, resulting in a resurgence of disease in this scenario. The emergence of antimicrobial resistance to cephalosporins at Vellore is consistent with available recent reports from other locations in India, where the ceftriaxone resistance rates ranged from 0% to 6%, and this emerging resistance is a matter of great concern.^11,35^

### Limitations in data and analyses.

The data described in the manuscript is from three different large tertiary care hospitals, which could not be considered as a representative of an Indian figure. In addition, the possibility of typhoid fever among non–school going children was not analyzed. Drinking water, usage of tap water for preparing artificial feed in children younger than 1 year, and contaminated street food in children older than 1 year could be the probable sources of infection, which were not studied. Another limitation is that, this is a laboratory-based retrospective analysis with limited blood culture data. However, an extensive literature search with detailed analysis was carried out to support the study findings.

This ecological analysis is limited to culture-confirmed typhoid from three large tertiary care facilities in urban and semi-urban settings. The national disease reporting system reports an increasing trend in clinician-diagnosed typhoid from 480,000 in 2001 to 1,800,000 in 2015. Although this increase may partly be explained by improvements in the reporting system, medical practitioners continue to treat acute febrile illness based on clinical and serological diagnosis as typhoid fever, with increasingly sophisticated antimicrobials.

Trends of culture-confirmed typhoid from tertiary care centers may be confounded by several factors, including prior antimicrobial therapy, differing health seeking practices, changes in guidelines for performing blood cultures, and changes in laboratory methods, including the use of resin-based automated blood culture systems.

The trends in contextual factors that might affect typhoid are derived from global databases that rely on data from government sources. In the absence of credible survey mechanisms that can reliably estimate these trends, the projections must be treated with caution. Furthermore, the observed trends provide summary measures for India, which can be very misleading, given the diversity and the size of the Indian population. All contextual factors are likely to change at different rates within population subgroups, and it would be reasonable to assume significant variations across regions and populations.

## CONCLUSION

Our findings from three Indian hospitals report a decline in culture-confirmed typhoid rates over the past 15 years at a faster pace than improvements in contextual factors. This declining trend may be a result of increasing per capita income, improved access to health care, early antibiotics, or other factors. However, typhoid fever clearly remains a public health problem in India, but the absence of comprehensive burden of disease data hampers decision making on interventional strategies. Increasing resistance to antibiotics may result in a resurgence of disease unless effective public health interventions are rapidly and appropriately deployed.
